# Prevalence of Depression and Psychological Distress and Perturbations of Cortisol Dynamics in Attendants of Hospitalized Patients: An Observational Pilot Study

**DOI:** 10.7759/cureus.12067

**Published:** 2020-12-13

**Authors:** Rimesh Pal, Naresh Sachdeva, Anil Bhansali, Akhilesh Sharma, Rama Walia

**Affiliations:** 1 Endocrinology, Post Graduate Institute of Medical Education & Research, Chandigarh, IND; 2 Psychiatry, Post Graduate Institute of Medical Education & Research, Chandigarh, IND

**Keywords:** ghq-12, patient attendant, psychological distress, depression, cortisol dynamics, phq-9

## Abstract

Background: Attendants of in-hospital patients are prone to undue stress resulting in depression, anxiety, melancholy and psychological distress. Hitherto available studies cater to attendants of patients admitted in critical care units and none have ventured to look into their cortisol dynamics. Herein, we have evaluated the magnitude of psychological distress and depression amongst ostensibly healthy attendants of non-critically ill patients and correlated them with cortisol dynamics.

Methods: Non-critically ill patients admitted to the general medicine ward were chosen by purposive sampling and one attendant was selected from each patient. Those with known risk factors, psychiatric illnesses, chronic drug intake, addictions, and overweight/obesity were excluded. Psychological distress and depression were assessed using the General Health Questionnaire 12-items (GHQ-12) and the Patient Health Questionnaire (PHQ-9), respectively. Morning plasma cortisol, late-night plasma cortisol (LNPC), late-night salivary cortisol (LNSC), urinary free cortisol (UFC), and plasma cortisol after overnight 1 mg dexamethasone administration were measured.

Results: After exclusion, 39 participants were recruited (M:F=2.3:1.6). The mean age was 34.1±11.4 years. The mean duration of stay in hospital ambience prior to recruitment was 16.2±1.2 days. Based on the PHQ-9 score, 55% of the participants had depression. Psychological distress prevailed in 13% of participants as per the GHQ-12 score. The median LNPC/LNSC was higher in participants with depression compared to those with no depression, however, there was no statistically significant difference. There was no significant correlation between GHQ-12/PHQ-9 scores and cortisol dynamics.

Conclusions: Although depression is prevalent in about half of the patient attendants, cortisol dynamics remain largely unaltered over a short period of two to three weeks.

## Introduction

Patients’ attendants are an indispensable adjunct to the hospital staff. They work just as much as the health-care professionals that expose them to significant occupational stress, which culminates in psychological disorders (depression, anxiety), fatigue, dissatisfaction, and maladaptive behaviors (substance abuse) [1]. However, an individual’s personality and coping skills ultimately decide whether and to what extent hospital ambience will lead to psychological distress. In general, females are more prone to develop psychological disorders than males [2], mostly because of the prevalent social barriers. 

Psychological health among attendants of in-hospital patients has never been investigated in detail. Few available studies have mostly concentrated on family members and relatives of patients admitted in the intensive care unit (ICU) and similar critical care settings [3-10]. All those studies have unanimously reiterated that family members of critically ill patients are fraught with stress, anxiety, depression, melancholy, and post-traumatic stress disorder (PTSD). However, studies performed in critical care settings might not reflect the actual psychological status in non-critical scenarios. ‘Fear of impending death’, one of the principal stressors in critical care settings might not be of that significant relevance in non-critical situations. 

Disturbances in the pituitary-hypothalamic-adrenal axis are common in chronic psychological disorders [11]. Cortisol excess is the most common anomaly, being reported in as many as 40-60% of drug-naïve patients of major depression [12]. Consequently, patients of melancholic depression suffer from numerous somatic sequelae that include osteoporosis, metabolic syndrome, coronary artery disease, and certain infectious and neoplastic diseases [13]. The other spectrum includes hypocortisolism that has been reported in 20-25% of patients with stress-related disorders like chronic fatigue syndrome, atypical depression, burn-out, and PTSD [14]. Altered cortisol dynamics have also been observed in professions associated with undue stress like night-shift workers and nursing staff [15-17]. However, abnormalities in the cortisol axis have hitherto never been studied amongst attendants of hospitalized patients. 

Hence, the present study was undertaken to evaluate the prevalence of psychological distress amongst apparently healthy attendants of patients admitted to the general ward of a tertiary care institution and correlate with various aspects of cortisol dynamics. 

## Materials and methods

A prospective observational study was conducted at the 120-bedded general medicine ward of a tertiary care government hospital in Northern India for over three months. Non-critically ill patients admitted in the ward were chosen every four weeks by systematic random sampling selecting every fifth bed from a random starting point. Thereafter, one family member/relative was selected from each chosen patient by simple random sampling using a random number table. Inclusion criteria included age more than 18 years, literate (able to read and write in Hindi), no prior history of any comorbid conditions with particular emphasis on diabetes mellitus, hypertension, coronary artery disease, connective tissue disease, rheumatological disease, bronchial asthma, chronic obstructive pulmonary disease, chronic liver or kidney disease or any known psychiatric disorder, no history of any chronic drug intake, no history of any addictions and body mass index (BMI) of 18.5-22.9 kg/m2. The participant must have been attending to his/her index patient in the hospital ward for at least two weeks before being included in the study. A baseline history was elicited from all the eligible participants to exclude any pre-existing psychiatric disorder. Written informed consent was obtained from all the participants. The study was approved by the Institute Ethics Committee, Post Graduate Institute of Medical Education and Research, India. 

Psychological health questionnaires

Psychological distress and depression were evaluated with well-validated questionnaires a couple of days before assessing cortisol dynamics. The primary investigator thoroughly explained the questionnaires and then the participants were asked to fill them up by themselves. 

Psychological distress was measured using the General Health Questionnaire short-form 12-items (GHQ-12) [18]. The standardized Hindi version was used for participant feasibility [19]. Each item was measured on a four-point Likert scale (one=better than usual, four=much worse than usual). As recommended, items were binary recoded (1-2=0, 3-4=1) before being summed. Individuals with scores of 7 and below were considered 'probable normal', scores from 8 to 15 as ‘probable psychological distress’ and more than 15 as ‘definite psychological distress’ [19].

Depression was assessed with the patient health questionnaire depression module (PHQ-9). It has been well validated in the general population and has been found to be useful in detecting major and subclinical depressive disorders [20]. The PHQ-9 scores each of the 9 Diagnostic and Statistical Manual of Mental Disorders, Fourth Edition (DSM-IV) criteria as ‘0’ (not at all) to ‘3’ (nearly every day). As for GHQ-12, the Hindi version of PHQ-9 was used. All nine individual scores were summed up to get the final score. A total score of 0-4 was regarded as having ‘no depression’, 5-9 as ‘mild depression’, 10-14 as ‘moderate depression’, and 15-19 as ‘moderately severe depression’.

Assessment of cortisol dynamics

Every participant had the following parameters measured as a part of a comprehensive assessment of cortisol dynamics: (a) Morning plasma cortisol, (b) late-night plasma cortisol (LNPC), (c) 24 hours urinary free cortisol (UFC), (d) late-night salivary cortisol (LNSC), and (e) 1-mg overnight dexamethasone suppression test (ONDST). 

Method of blood sample collection for the estimation of cortisol

A morning plasma cortisol sample was collected exactly between 0800-0900 hours. For late-night plasma cortisol, a blood sample was drawn at 2300 hours in the awake state. All the blood samples were centrifuged immediately, plasma separated and stored at -20°C. Samples were transported to the laboratory in an ice pack for processing. ONDST was performed by orally administering a 1-mg tablet of dexamethasone at 2300 hours with a blood sample for cortisol collected at 0800 hours the next day. 

Method of saliva collection for the estimation of LNSC

On the day of sample collection, participants were instructed to eat at least two hours prior to collection and not to brush their teeth for at least four hours prior to the test. Salivate (SalivaBio Oral Swab from Salimetrics, Carlsbad, CA) was given and patients were asked to moisten it with saliva by keeping it in their mouth for three to five minutes. Saliva was then collected in a plain vial at 2300 hours. Samples were kept in a refrigerator and transported to the laboratory the next day. 

Method of urine collection for the estimation of 24 hours UFC

Urine was collected for 24 hours for three consecutive days. Participants were instructed to void urine at 0900 hours on the first day of collection and collect all the urine in a clean container until the next morning at 0900 hours and store it in the ward refrigerator. The total volume was measured and 30 mL of sample was sent to the laboratory for cortisol estimation. All three samples were stored at -20°C for subsequent processing. The average of the three values was considered as the 24 hours UFC of the participant. It was ensured that ONDST be performed after completion of urine collection as prior administration of dexamethasone could lead to suppression of the hypothalamic-pituitary-adrenal axis and falsely low UFC values.

Assays used

Plasma and salivary cortisol were measured using electrochemiluminescence immunoassay (ECLIA) (ELECSYS-2010, Roche Diagnostics, Basel, Switzerland). UFC was measured using the dichloromethane extraction method. Inter- and intra-assay coefficients of variation were 1.4-2.8%, 1.5-14.2%, and 1.8-4.7% for plasma cortisol, LNSC, and UFC, respectively. 

The cut-offs used to define hypercortisolism were based on a study performed by Jarial et al. in 24 patients of Cushing’s syndrome and 16 patients of pseudo-Cushing's states [21]. Values that had the maximum sensitivity in diagnosing hypercortisolism were considered. Accordingly, a cut-off of 213.67 nmol/L for morning plasma cortisol and 116.04 nmol/L for LNPC was chosen (sensitivity being 100% for each). LNSC and 24 hours UFC had 100% sensitivity at a value of 4.95 nmol/L and 76.7 μg/day, respectively [21). The cut-off for ONDST was set at 50 nmol/L (sensitivity 100%). 

Statistical analysis

Statistical Package for the Social Sciences (SPSS) version 23 (IBM, SPSS Inc, Chicago, IL) was used for the statistical analyses. The normality of data was checked using the Shapiro-Wilk method. Normally distributed data were expressed in terms of mean ± standard deviation (SD). Non-parametric data, on the other hand, was expressed in terms of the median (interquartile range). Cortisol levels were correlated with GHQ-12 and PHQ-9 scores using Pearson/Spearman correlation (based on normality of data). Between-group comparisons were made by the Mann-Whitney *U* test or Independent Samples *t*-test. 

## Results

A total of 50 patient attendants were initially recruited and subsequently screened for the presence of any of the exclusion criteria. Eleven of them met one or more of the exclusion criteria leaving behind 39 participants for final analysis (four had type 2 diabetes mellitus, two were on anti-psychotic medications, three were chronic alcohol consumers, two were obese with BMI > 28 kg/m2). Amongst these 39 final participants, there were 23 males and 16 females. The mean age of the participants was 34.1 ± 11.4 years. The mean BMI was 21.2 ± 1.6 kg/m2. When the relationship of the attendants’ to the index patient was considered, most of them were found to be children (28.2%), parents (23%), spouse (18%), siblings (12.8%), or others (18% namely cousin, nephew, neighbor, son-in-law, brother-in-law). The mean duration of stay in the hospital ambience prior to being recruited in the study was 16.2 ± 1.2 days.

The mean GHQ-12 score of the participants was 4.8 ± 2.3. Among them, five participants qualified as having 'probable psychological distress', and 34 were labeled 'probable normal'. None of the participants had 'definite psychological distress'. The median PHQ-9 score of the participants was 5.0 (IQR 2.0-9.0). On the basis of the PHQ-9 score, 13 participants (33%) had 'mild depression', five (13%) had 'moderate depression', and three (7.6%) had 'moderately severe depression'. All patients with a GHQ-12 score of more than seven had a PHQ-9 score of more than four. There was no statistically significant difference in either GHQ-12 or PHQ-9 scores between men and women.

Cortisol dynamics among the participants

The overall cortisol parameters of all the 39 participants have been summarized in Table 1.

**Table 1:** Cortisol parameters of all the participants (N=39). SD: standard deviation; IQR: interquartile range; LNPC: late-night plasma cortisol; LNSC: late-night salivary cortisol; UFC: urinary free cortisol; ONDST: overnight dexamethasone suppression test

Cortisol Parameter	Mean ± SD OR Median (IQR)
800 hours cortisol	267.0 ± 89 nmol/L
LNPC	89.0 nmol/L (IQR: 48.6-166.0)
LNSC	5.0 ± 2.1 nmol/L
24 hours UFC	31.6 μg/day (IQR: 12.8-87.5)
Plasma cortisol after ONDST	23.5 nmol/L (IQR: 20.2-29.1)

There were no significant differences in cortisol dynamics regarding gender, although the median LNPC in women was much higher than in men (163.5 nmol/L vs. 73.0 nmol/L, p=0.06). Amongst the 39 participants, 28 (72%) had a morning plasma cortisol value above the stipulated cut-off of more than 213.67 nmol/L. Eighteen participants (46%) had an LNPC above the preset cut-off of 116.04 nmol/L. Seventeen of them (43.5%) had high morning cortisol values as well. Elevated LNSC and UFC were seen in 16 (41%) and 12 (31%) participants, respectively. Only two individuals had elevated morning plasma cortisol, LNPC, LNSC, and UFC. None had plasma cortisol of more than 50.0 nmol/L following an ONDST. 

Correlation between cortisol dynamics with psychological health scores

There was no statistically significant correlation between GHQ-12 scores and morning plasma cortisol (r= -0.158, p=0.338), LNPC (rs= 0.077, p=0.643), LNSC (r= -0.089, p=0.558), UFC (rs= 0.002, p=0.990) or plasma cortisol following ONDST (rs= 0.037, p=0.824). Similarly, PHQ-9 scores did not show any statistically significant correlation with any of the cortisol parameters [morning plasma cortisol (rs= 0.102, p=0.535), LNPC (rs= 0.103, p=0.531), LNSC (rs= 0.150, p=0.363), UFC (rs= -0.151, p=0.360), plasma cortisol following ONDST (rs= 0.172, p=0.296)]. 

The median GHQ-12 scores for participants with morning plasma cortisol above and below 213.67 nmol/L were 5.0 and 6.0, respectively. The corresponding PHQ-9 scores were 5.5 and 5.0, respectively. There was, however, no statistically significant difference in either of the two scores in both the sub-groups. Similarly, the median GHQ-12 scores for participants with LNPC above and below 116.04 nmol/L were 5.5 and 5.0, respectively. The corresponding PHQ-9 scores were 6.0 and 5.0, respectively. There was no statistically significant difference in either of the two scores in between the sub-groups. 

The morning plasma cortisol and LNPC values were divided into quartiles. There was no statistically significant difference in GHQ-12 or PHQ-9 scores among them.

**Figure 1:**
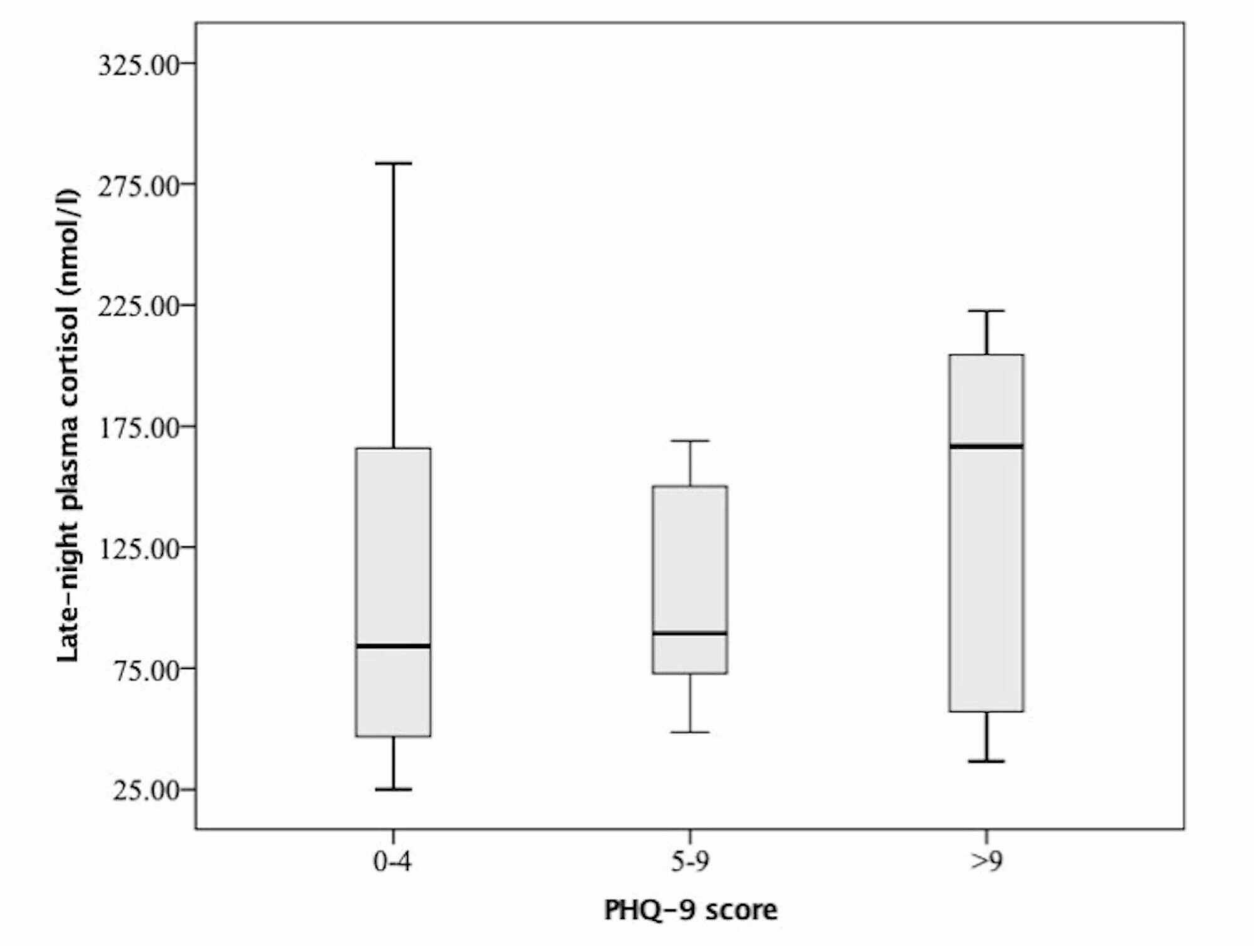
Box and whisker plot showing late-night plasma cortisol in participants with no depression (PHQ-9 score 0-4), mild depression (PHQ-9 score 5-9), and moderate/moderately severe depression (PHQ-9 score > 9).

Based on PHQ-9 scores, the median morning plasma cortisol values in those with 'no depression' (score 0-4), 'mild depression' (score 5-9), and 'moderate/moderately severe depression' (score ≥ 10) were 258.2 nmol/L, 249.5 nmol/L, and 338.5 nmol/L, respectively. Corresponding values for LNPC were 84.1 nmol/L, 89.4 nmol/L, and 166.8 nmol/L (Figure 1).

**Figure 2:**
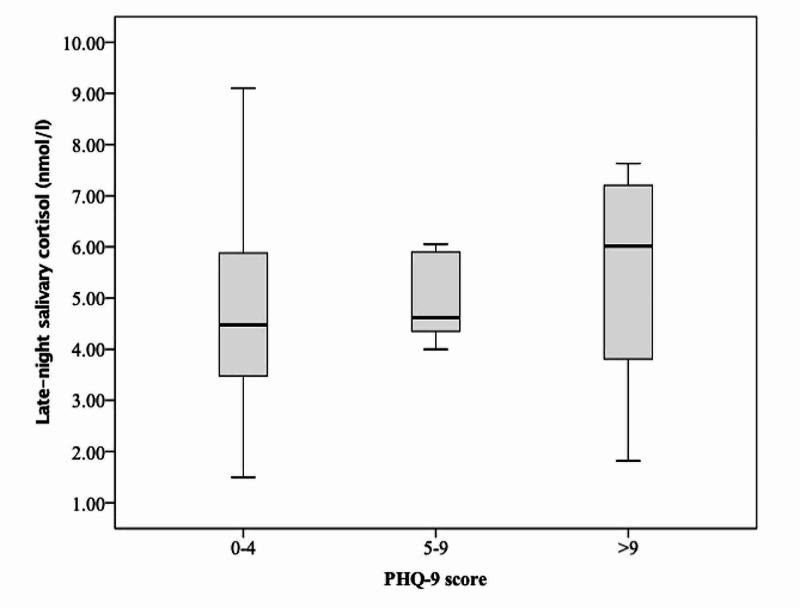
Box and whisker plot showing late-night salivary cortisol in participants with no depression (PHQ-9 score 0-4), mild depression (PHQ-9 score 5-9), and moderate/moderately severe depression (PHQ-9 score > 9).

LNSC values in the three groups were 4.4 nmol/L, 4.6 nmol/L, and 6.0 nmol/L, respectively (Figure 2).

There was no statistically significant difference amongst the three groups in morning plasma cortisol, LNPC, LNSC, UFC, or ONDST values. 

Similarly, based on GHQ-12 scores, the median morning plasma cortisol values in those with ‘probable normal’ vs. those with ‘probable psychological distress’ were 254.6 nmol/L and 209.5 nmol/L, respectively. Median values for LNPC were 108.0 nmol/L and 78.6 nmol/L, respectively; those for LNSC were 4.8 nmol/L and 4.4 nmol/L, respectively. There was no statistically significant difference among the two groups in morning plasma cortisol, LNPC, LNSC, UFC, or ONDST values. 

## Discussion

The above study highlights that depression is quite prevalent among attendants of patients admitted in the general medicine ward, affecting almost 55% of the participants based on PHQ-9 score. Median morning plasma cortisol, LNPC, and LNSC were numerically higher in those with ‘moderate/moderately severe depression’ as compared to those with ‘mild depression’, however, there was no statistically significant correlation between cortisol dynamics and PHQ-9 scores.

Hospital-stay without actual hospitalization adversely affects the psychological well being contributing to stress, anxiety, depression, and maladaptive behaviors. In a study conducted among 150 patient attendants, 92.6% and 76% of the participants had depression and anxiety, respectively [1]. Hamilton Depression Rating Scale (HDRS) was used to screen participants for depression. Instead, in our study, we used the PHQ-9 questionnaire and found that 55% of our participants had depression. Although there is a positive correlation between PHQ-9 and HDRS [22], the latter is psychometrically and conceptually flawed, as it places more emphasis on insomnia than on feelings of hopelessness, self-destructive thoughts, and suicidal ideations. Most of the scale items are poor contributors to the measurement of depression severity [23]. PHQ-9, on the other hand, explicitly addresses all the nine Diagnostic and Statistical Manual of Mental Disorders, Fourth Edition (DSM-IV) criteria for depression [24]. It has a sensitivity of 77%, a specificity of 94%, and a positive predictive value as high as 85-90% in screening for depression [25]. 

In a study performed amongst attendants of patients admitted in ICU, the prevalence of depression was 68% using the Hospital Anxiety and Depression Scale (HADS) [7]. This data seems to be much more realistic than that published by Rajput et al. and is concordant with our findings. The HADS, unlike the PHQ-9 scale, has the added advantage of assessing both depression (HADS-D) and anxiety (HADS-A). Although the agreement between HADS-D and PHQ-9 is moderate, studies have shown that the identified prevalence of depression is similar when using the two scales. However, as the PHQ-9 scale is derived from DSM-IV, it has a greater appeal, at least in research settings [26]. A study of 501 patients showed that PHQ was superior to HADS [24].

The other psychological health questionnaire used in our study was the GHQ-12. Only 13% of our participants were found to have ‘probable psychological distress’ while the rest were categorized as being ‘probable normal’. This figure seems to be too low. To the best of our knowledge, GHQ-12 has hitherto never been used in the past to assess psychological distress amongst patient attendants. Our attempt at using GHQ-12 as a measure of psychological distress could have led to fallaciously low results. It is likely because of the inherent response bias on the negative items. Moreover, the PHQ-9 is known to function better than the GHQ-12 as a screening tool for depression, hence the results derived from each of the two scales are better left uncompared [27]. Large-scale observational studies comparing GHQ-12 scores of patient attendants and the general population need to be undertaken. 

Our study did not find any correlation between the participants’ psychological health, as measured by GHQ-12/PHQ-9 and cortisol dynamics. Yet again, this was a novel attempt and was based on the premise that cortisol dynamics get altered in shift workers and nursing professionals [15-17]. However, the fact that needs to be considered in all these studies is the population being investigated. The participants in all these studies had been exposed to psychological distress for a long time instead of our volunteers who had been visiting and taking care of their patients for a mean duration of only 16.2 days. Other than subtle perturbations in the circadian rhythm of cortisol secretion, it would be too early to expect major fluctuations in cortisol dynamics over a period of two weeks. LNPC and LNSC were numerically higher in those with depression as opposed to those with no depression; in fact, those with ‘moderate/moderately severe depression’ had higher values than those with ‘mild depression’, however, there were no statistically significant differences in between these three groups likely owing to the small sample size. Rather than applying specific cut-offs to define cortisol excess, comparison of mean cortisol values of our participants with an age and sex-matched control population might have been more rewarding. In addition, existing literature supports the fact that cortisol levels are elevated in patients with major depressive disorder with little or no data in patients with mild depression [28]. As most of our participants who qualified as having depression as per the PHQ-9 scale had the milder form and only 8 participants had major depression (defined as PHQ-9 ≥ 10), the cortisol values were not statistically different from those with no depression. Lastly, other aspects of psychological health, namely, stress, anxiety, and burnout symptoms were not assessed in the present study as they have been known to be independently associated with cortisol levels [16,29]. 

Our study does have certain limitations. Firstly, the sample size is small. However, the fact that we had planned a preliminary observational study does support our endeavor. Secondly, we do not have a control group comprising of healthy age- and sex-matched subjects residing in the community. A large community-based survey in Southern India had shown that the prevalence of depression was only 15.1% [30]. Hence, we can conclude that a prevalence of 55%, as seen in the present study, is definitely higher than the general population. Thirdly, in comparison with a study population simultaneously recruited from a critical care setting would have provided comparative data. Lastly, cortisol dynamics were evaluated at one single time point. A serial assessment might have helped delineate the chronological alternations in cortisol dynamics. 

## Conclusions

Psychological health is adversely affected in attendants of patients admitted in a general hospital ward. Depression is prevalent in about half of the attendants. Cortisol dynamics, namely LNPC and LNSC, although numerically higher in depressed attendants, do not differ statistically from those without depression. A large-scale study with a properly recruited control group might prove worthwhile in unearthing the nitty-gritty of cortisol dynamics in patient attendants.
